# Clinical features and genetic analysis of Dandy-Walker syndrome

**DOI:** 10.1186/s12884-023-05367-1

**Published:** 2023-01-18

**Authors:** Yanmei Sun, Tao Wang, Ning Zhang, Pingping Zhang, Yali Li

**Affiliations:** 1 Department of Reproductive Genetics, Hebei General Hospital, Shijiazhuang, 050051 China; 2 The Second People’s Hospital of Liaocheng, Liaocheng, 252600 China

**Keywords:** Dandy-Walker syndrome, Prenatal diagnosis, SNP-array, Chromosomal abnormalities

## Abstract

**Background:**

Dandy-Walker syndrome (DWS) is a rare congenital malformation of the central nervous system (CNS), characterized by underdevelopment or dysplasia of the cerebellar vermis, expansion of the fourth ventricle and posterior fossa cistern. The incidence is aboutapproximately 1/25000–1/35000. At present, the etiology and pathogenesis of DWS are not completely clear. It is mostly considered to be a multifactorial genetic disease that is related to both genetic factors and environmental factors. There is no large sample size analysis of the chromosomal profile of DWS up to now. This study aims to provide clinical reference for prenatal diagnosis via summarizing the clinical features and pregnancy outcomes of Dandy-Walker syndrome.

**Methods:**

A total of 76 cases of foetal Dandy-Walker syndrome out of 19,506 pregnant women underwent cordocentesis or amniocentesis for genetic detection. Rapid prenatal karyotyping, single nucleotide polymorphism array (SNP-array) and BACs-on-Beads™ (BoBs) were performed for prenatal genetic diagnosis. The results of ultrasonography, genetic analysis and pregnancy outcome were recorded.

**Results:**

Of the 76 cases, 19 were isolated DWS, while 57 cases were accompanied by other ultrasound-visible abnormalities. Ultrasound abnormalities of the CNS were most frequently observed, accompanied by DWS. Twenty-five out of 76 cases had chromosomal abnormalities, and the rate of chromosomal abnormalities increased in pregnant women of advanced maternal age or in combination with other ultrasound abnormalities. Of the 19 cases in the isolated DWS group, nine pregnant women chose to terminate the pregnancy, while seven cases continued the pregnancy and all infants were normal. Among the 57 pregnant women with pathological ultrasound manifestations other than foetal DWS, 44 chose to terminate the pregnancy, while 12 cases continued the pregnancy. Further follow-up revealed one newborn with postnatal neurodevelopmental delay. A female term neonate presented with very severe sensorineural deafness, and an infant died 7 days after birth with abnormal development of multiple organs.

**Conclusions:**

Pregnant women with DWS in foetal ultrasonic examination should be offered a careful and comprehensive foetal ultrasound scan and further prenatal genetic testing including karyotype analysis and SNP-array. The prognosis of the foetus without chromosomal aberration is good in isolated DWS pregnancies but poor in nonisolated DWS pregnancies.

## Introduction

Dandy-Walker syndrome (DWS) is a rare congenital malformation of the central nervous system (CNS), characterized by underdevelopment or dysplasia of the cerebellar vermis, expansion of the fourth ventricle and posterior fossa cistern [1]. The incidence is approximately 1/25000–1/35000 [2, 3]. Taking the development of the cerebellar vermis as a standard, it is divided into three types: Dandy-Walker malformation, Dandy-Walker variant and simple posterior fossa cistern widening [4]. Clinically, it can appear as a single malformation, or it can be accompanied by malformations in other parts, including malformations in the CNS and non-CNS.

At present, the etiology and pathogenesis of DWS are not completely clear. It is mostly considered to be a multifactorial genetic disease that is related to both genetic and environmental factors. The chromosomal abnormalities increased when accompanied by other malformations [5]. DWS is usually confirmed by magnetic resonance imaging or pathological examination after delivery, but the prognosis of the foetus is poor, and early diagnosis is often advocated. Due to the rarity of DWS and heterogeneity of clinical manifestations, previous studies of the disease are mostly case reports or literature reviews. The current understanding of its genetic etiology is limited, and it is very challenging to conduct genetic counselling after DWS is diagnosed. In this study, the clinical data of 76 patients with DWS were summarized (not including simple posterior fossa cistern widening), hoping to provide references for further elucidating the pathogenesis and prenatal diagnosis of DWS.

## Patients and methods

From January 2009 to March 2021, there were 19,506 pregnant women who underwent prenatal genetic diagnosis with indications for genetic amniocentesis or cordocentesis under ultrasound guidance in the Department of Reproductive Genetic Family of Hebei General Hospital. A total of 76 pregnant women with foetal DWS underwent cordocentesis or amniocentesis. The ultrasound diagnostic criteria for DWS were that the foetal bilateral cerebellar hemispheres were not completely separated, the cerebellar vermis was missing to varying degrees, the fourth ventricle was dilated, and the abnormally enlarged posterior fossa cistern was penetrated. The width of the posterior foot of the body of the lateral ventricle was greater than 10 mm defined as ventricular enlargement. The results of intracranial and extracranial ultrasonography were tabulated, and the results of genetic analysis of the investigated cases were recorded. All examinations were performed by experienced sonographers using a 5 MHz transducer (IU22, Philips; VOLUSON E8, GE). Rapid prenatal karyotyping was performed for all samples, while only 22 cases performed single nucleotide polymorphism array (SNP-array) and 4 cases performed BACs-on-Beads™ (BoBs) due to high cost.

We used the Statistical Package for the Social Sciences Version 26.0 (SPSS, IBM, Chicago, IL, USA) to perform statistical analyses. The *χ2* test was used to compare the differences between groups. Differences were considered significant at *P < 0.05*.

## Results

**Fig. 1:**
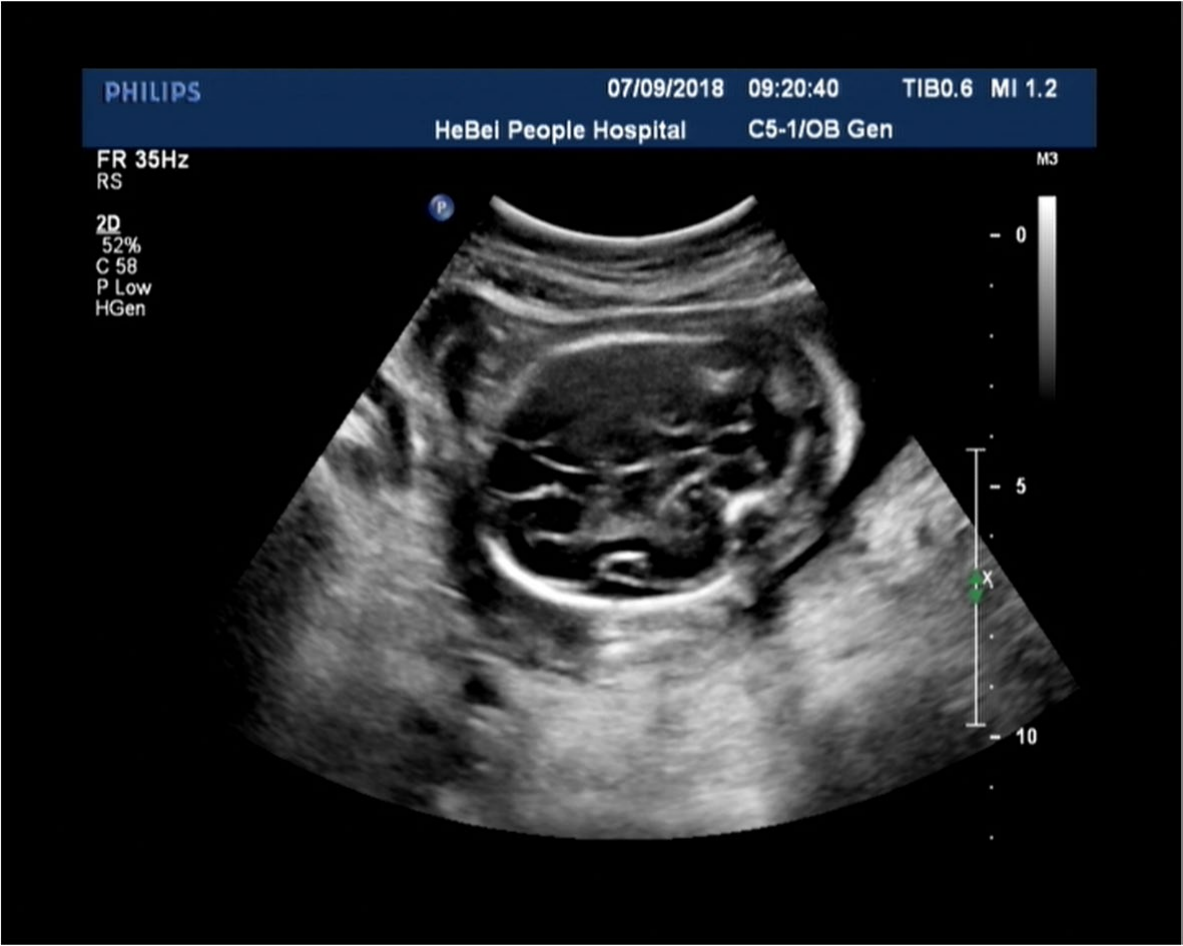
The ultrasound example of a DWS foetus. It shows a partial absence of the vermis, dilation of the fourth ventricle, and an enlarged posterior fossa

Of the 76 pregnant women with foetal DWS (Fig. 1) undergoing invasive sampling procedures, 38 underwent amniocentesis and 38 underwent cordocentesis. The mean gestational age of pregnant women who underwent paracentesis was 25.57 ± 5.97 weeks in isolated group and 25.13 ± 4.97 weeks in nonisolated group respectively, and the mean maternal age was 28.21 ± 4.93 years old in isolated group and 28.35 ± 5.54 in nonisolated group respectively. (Table 1).

**Table 1:** Basic characteristics of 76 fetuses with Dandy-Walker syndrome

	Isolated group	Nonisolated group
Mean age	28.21 ± 4.93	28.35 ± 5.54
Average gestational week	25.57 ± 5.97	25.13 ± 4.97
Type of sample		
Amniotic fluid	10	28
Umbilical vein blood	9	29
Testing items		
karyotype	14	36
Karyotype+BoBs	1	3
Karyotype+SNP-array	4	18
Total	19	57

Of the 76 cases, 19 (25.0%) were isolated DWS, while 57 (75.0%) were accompanied by other ultrasound-visible abnormalities. Ultrasound abnormalities of the CNS were the most frequently observed abnormalities associated with DWS, accounting for 29.1%, and the most common CNS anomaly was ventricular widening accounting for 19.7%. The most common non-CNS abnormality was the cardiovascular system, accounting for 27.6% (21/76), followed by abnormalities in the digestive system, limbs, renal system, face and neck. Other concomitant pathological ultrasound findings may also include polyhydramnios, single umbilical artery, widened renal pelvis, obstruction of the digestive tract, polydactyl, etc. (Table 2).

**Table 2:** Associated CNS and extra-CNS abnormalities with DWS

Anomaly	n	%^a^
*Central nervous system*	25	29.1
Ventriculomegaly	15	19.7
Choroid plexus cyst	4	5.3
Abnormal cavum of setumpellucidum	4	5.3
Blake’s pouch cyst	1	1.3
Meningocele	1	1.3
*Cardiovascular system*	21	27.6
Ventricular septal defect	10	12.2
Persistent left superior vena cava	3	3.9
Ebstein anomaly	1	1.3
Aortic riding span	1	1.3
Uneven heart development	1	1.3
Small internal diameter of the aortic arch	1	1.3
Complete endocardial cushion absence	1	1.3
Complex cardiovascular developmental malformations	3	3.9
*Digestive System*	10	13.2
Gallbladder enlargement	4	5.3
Small or undetected stomach vesicles	3	3.9
Digestive tract obstruction	3	3.9
*Extremity*	8	10.5
Abnormal posture	3	3.9
Shortness	2	2.6
Polydactyly	2	2.6
Abnormal morphology	1	1.3
*Kidney system*	6	7.9
Hydronephrosis	5	6.6
Enlarged Kidney	1	1.3
*Face & Neck*	6	7.9
Neck cyst	3	3.9
Nasal bone dysplasia	3	3.9
*Other*		
Single umbilical artery	7	9.2
Polyhydramnios	7	9.2
Nuchal translucency thickening	4	5.3
Intrauterine growth restriction	2	2.6
Umbilical hernia	2	2.6
Visceral version	1	1.3
Umbilical cord cyst	1	1.3
Diaphragmatic hernia	1	1.3

^a^The percentage of associated anomalies in total 76 fetuses

*Note*: Repeat counts if ≥2 ultrasound indices are abnormal in the same fetus

Among the 76 foetuses who underwent karyotype examination, 25 (32.9%) had chromosomal abnormalities, including 4 (21.1%) in the isolated group and 21 (36.8%) in the nonisolated group. Of the 76 cases, 17 (22.4%) had chromosomal numerical abnormalities, and 8 (35.16%) had chromosomal structural abnormalities; trisomy 18 was the most noted. There was no statistically significant difference between the isolated group and the nonisolated group in chromosomal numerical abnormalities (χ2 = 0.227, *P* = 0.634) or chromosome structure abnormalities (χ2 = 0.186, *P* = 0.666). (Table 3).

**Table 3:** Chromosome abnormalities found in fetuses with or without other pathological ultrasound findings [n, (%)]

Chromosome abnormality	Total [n^a^, (%)]	Isolated DWS [n^b^, (%)]	Nonisolated DWS [n^c^, (%)]
*Numerical abnormalities*	17 (22.4)	3 (15.8)	14 (24.6)
Trisomy-18	8 (10.5)	2 (10.5)	6 (10.5)
Trisomy-21	5 (6.6)	0	5 (8.8)
Trisomy-13	3 (3.9)	0	3 (5.3)
69, XXX	0	1 (5.3)	0
*Structural abnormalities*	8 (10.5)	1 (5.3)	7 (12.3)
46, Xn, add (8) (p11)	1 (1.3)	0	1 (1.8)
46, Xn, der (8) t(7;8) (p21; q43)	1 (1.3)	0	1 (1.8)
46, Xn, del (1) (q42)	1 (1.3)	0	1 (1.8)
46, Xn, der (8) (8pter-8q24::12p10-12qter), i(12) (p10)	1 (1.3)	0	1 (1.8)
46, Xn, rec (3) dup (3q) inv.(3) (p25q23)	1 (1.3)	0	1 (1.8)
46, Xn, der (2) t(2; 11) (q35; q13)	1 (1.3)	0	1 (1.8)
47, XN, +Mar	1 (1.3)	0	1 (1.8)
46, XN, t (10; 21) (p10; q10)	1 (1.3)	1 (5.3)	0
Total	25 (32.9)	4 (21.1)	21 (36.8)

^a^The percentage of associated anomalies in total 76 fetuses

^b^The percentage of associated anomalies in total 19 fetuses

^c^The percentage of associated anomalies in total 57 fetuses

**Fig. 2:**
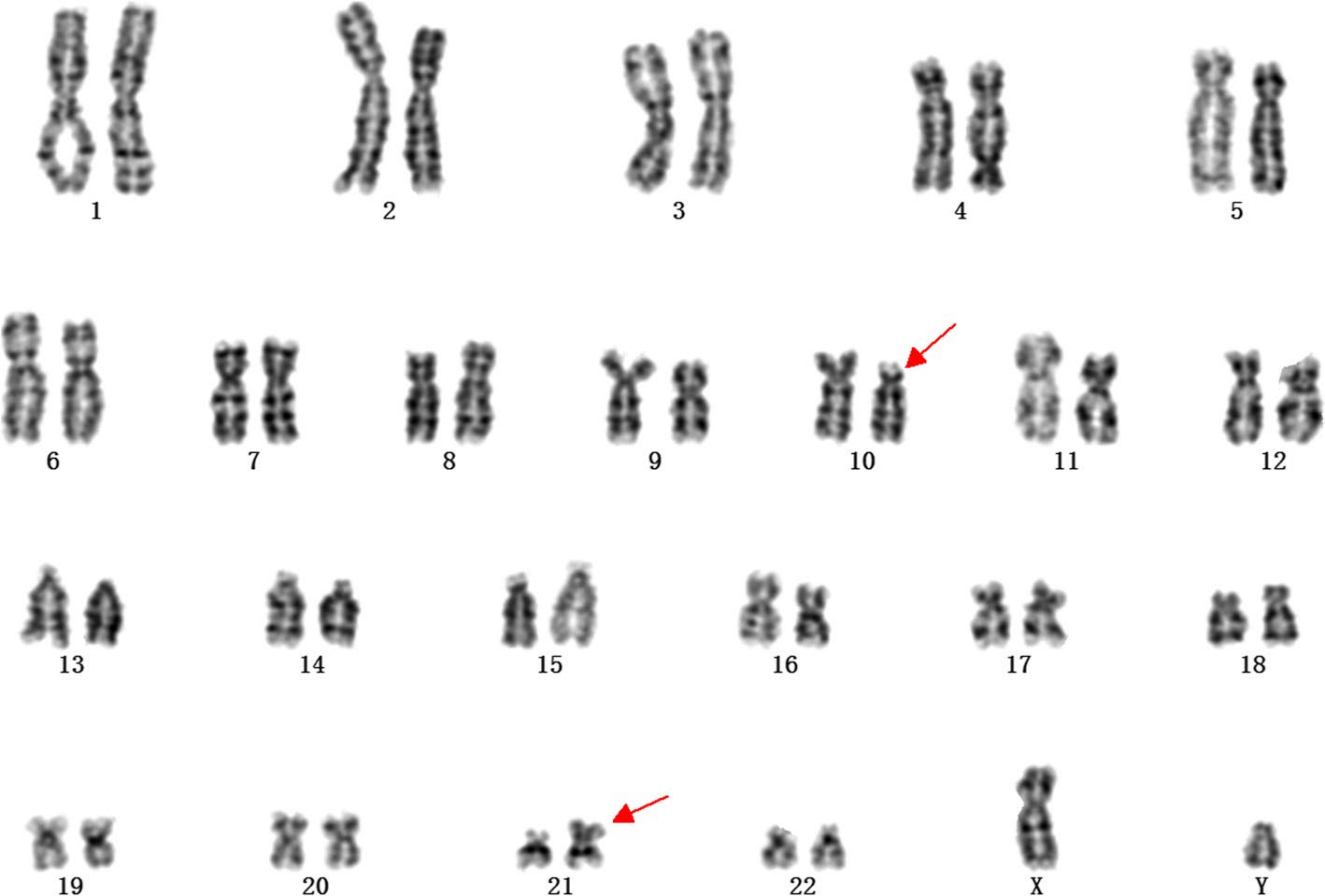
The karyotype result of case6 in Table 4. It shows 46,XN,t (10;21)(p10;q10)

**Fig. 3:**
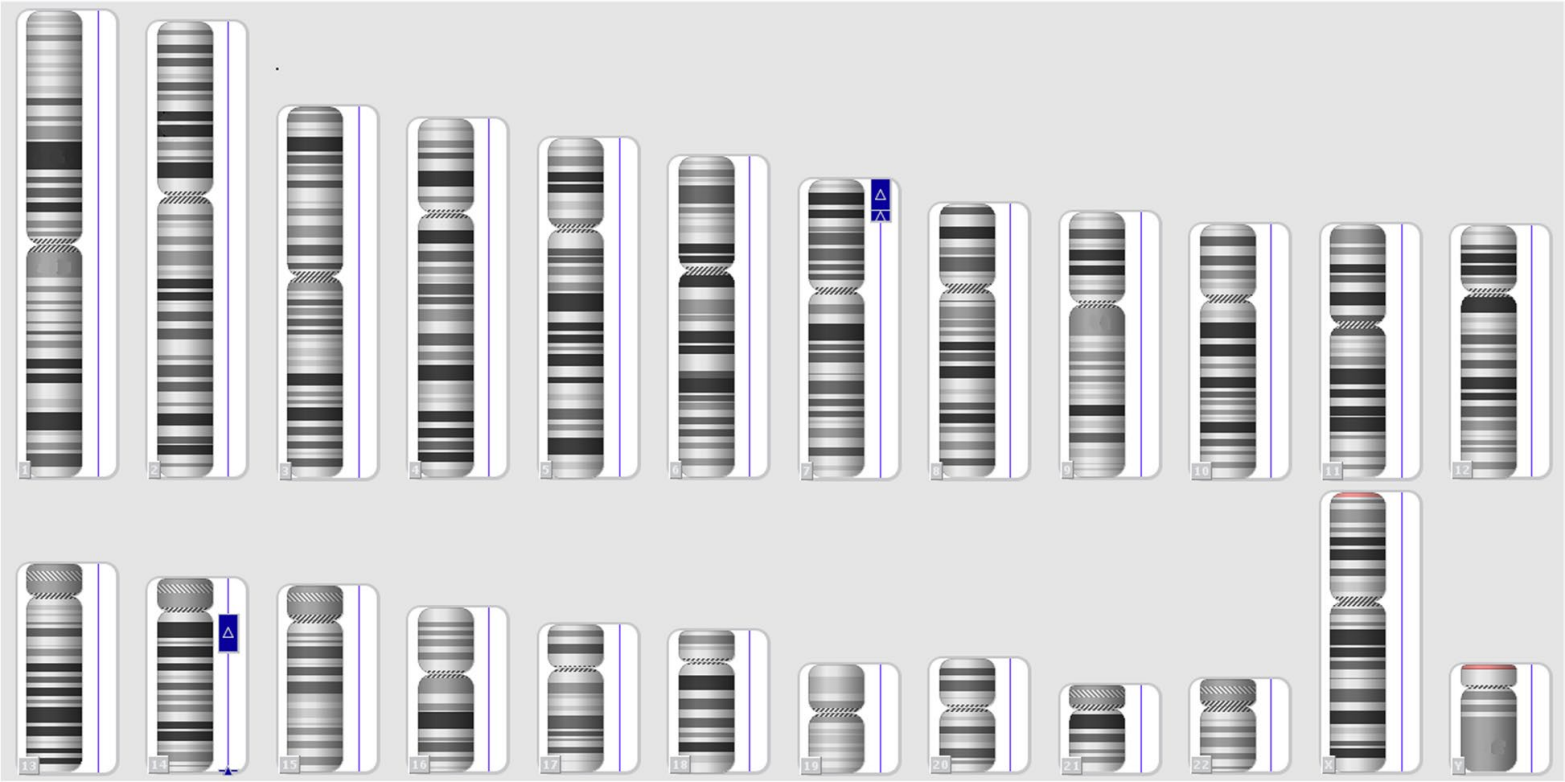
The SNP-array Karyoview of case3 in Table 4. It shows a duplication on 14q11.2q21.1 and a duplication on 7p22.3p15.3

A total of four foetuses underwent BoBs examination, which were in accordance with the results of karyotype. A total of 22 foetuses (28.9%, 22/76) underwent both karyotype and SNP-array analysis, as shown in Table 3. Karyotype analysis and chromosomal SNP-array were not always concordant with each other. SNP-array can identify microduplications or microdeletions smaller than 5 Mb, however, it cannot identify balanced translocation (Table 4). The karyotype result of case6 in Table 4 (Fig. 2) and The SNP-array result of case3 in Table 4 (Fig. 3).

**Table 4:** Chromosome abnormalities in karyotyping and SNP-array analysis

	Karyotype	SNP-array
Case1	46,Xn,der(8)(8pter-8q24::12p10-12qter),i(12)(p10)	arr [hg19]12p13.3p11.1 (173786–34,835,641)*3,15q11.2)(2,770,421–23,276,605)*1
Case2	Trisomy 18	arr [hg19]18p11.32q23(136227–78,013,728)*3
Case3	47,Xn,+Mar	arr [hg19]7p22.3p15.3 (43376–22,353,054) × 3 arr [hg19]14q11.2q21.1 (20516277–40,785,508) × 3
Case4	69,XXX	arr [hg19](1–22,X)*3
Case5	Normal	arr [hg19]7p22.2p21.2 (3720000–15,180,000)*3
Case6	46,Xn,t(10;21)(p10;q10)	Normal
Case7	46,Xn,t(7;8)(p21;q43)	Normal
Case8–22	Normal	Normal

There were four (21.1%, 4/19) chromosomal abnormalities in the isolated group, while eight cases in the nonisolated group had one other ultrasound abnormality, seven cases in the nonisolated group had two other ultrasound abnormalities, and seven cases in the nonisolated group had three other ultrasound abnormalities, and the comparison between groups suggested a statistically significant difference between groups (χ2 = 7.757, *P* = 0.046). (Table 5).

**Table 5:** The relationship between the number of accompanying ultrasound abnormalities and chromosomal abnormalities

*Ultrasound abnormalities*	Total	Chromosome abnormality	%	*X*^*2*^	*P*值
*Isolated Dandy-Walker*	19	4	21.1		
*Associated with 1 anomaly*	30	8	26.7		
*Associated with 2 abnormalities*	17	7	41.2		
*Associated with ≥ 3 abnormalities*	10	7	70.0	7.757	0.046

*Note*: Including 1 case with normal karyotype but abnormal SNP-array

Among the 76 cases, there were 9 women of advanced maternal age, of which 8 (88.9%) had chromosomal abnormalities, while 26.9% were young women. The difference was statistically significant between the two groups (χ2 = 10.945, *P* = 0.001). (Table 6).

**Table 6:** Association of advanced maternal age with chromosomal abnormalities

Maternal age	Total	Chromosome abnormality^a^	%	*X*^*2*^	*P*值
< 35 years	67	18	26.9		
≥35 years	9	8	88.9		
Total	76	26	34.2	10.945	0.001

^a^*Note*: Including 1 case with normal karyotype but abnormal SNP-array

The prognosis of the 76 cases was followed-up; however, 3 cases in the isolated group and 1 case in the nonisolated group were lost. Of the 19 cases in the isolated group, 9 pregnant women chose to terminate the pregnancy (including 2 cases with trisomy 18 or triploid); all 7 cases that continued the pregnancy survived through the pregnant period, and all infants were normal.

Among the 57 pregnant women with pathological ultrasound manifestations other than foetal DWS, 44 chose to terminate the pregnancy due to foetal chromosomal abnormalities or severe multiple structural abnormalities. Eight of the 44 induced foetuses exhibited visible malformations, such as facial malformation, cranial malformation, or abnormal morphology of both lower limbs. Among the 12 cases that continued the pregnancy, further follow-up revealed one newborn with postnatal neurodevelopmental delay, but its prenatal and postnatal MRI showed normal results. A female term neonate who presented with very severe sensorineural deafness was found after birth, but no carriage of the deafness gene required cochlear hearing assistance. One infant died 7 days after birth with abnormal development of multiple organs (Table 7).

**Table 7:** Prognosis of fetuses with DWS

Group	Total(n)	lost to follow-up	Induced abortion	Live birth	Normal postnatal development
Isolated DWS	19	3	9	7	7
Non-isolated DWS	57	1	44	12	8

## Discussion

Ultrasound is still the most effective method for prenatal diagnosis of DWS, which can be found during prenatal ultrasound screening in the second trimester. Previous studies believe that DWS should not be diagnosed prematurely, because the cerebellar vermis develops from top to bottom at 17–18 weeks [5]. In this study, a pregnant woman underwent genetic amniocentesis at 16^+ 3^ weeks of gestation due to foetal lymphatic hygroma of the neck and DWS.

In this study 57 cases were found to be accompanied by other ultrasound-visible abnormalities, which accounted for 75.0%, and the rate of chromosome abnormality was 32.9% (excluding one showing 46, Xn, t (7; 8) (p21; q43) in karyotyping but normal in SNP-array).

DWS is often accompanied by ultrasonographic structural abnormalities. In this study, ultrasound abnormalities in the CNS were the most common, followed by abnormalities of the cardiovascular system, indicating that systematic prenatal ultrasonography should be performed when DWS is detected in prenatal ultrasound scans, focusing on the CNS and cardiovascular system [6]. Correspondingly, when discovering other system abnormalities, we should also pay attention to the cerebellum and posterior fossa [7].

The study showed that DWS was associated with chromosomal aberrations, especially aneuploidy, which is similar to previous studies [7, 8], and it showed that the number of foetal ultrasound abnormalities and advanced maternal age appeared to be risk factors for chromosomal abnormalities in foetuses with DWS. Therefore, when DWS is found in prenatal examination, foetal chromosomes should be checked [8]. However, conventional chromosome analysis can only detect chromosome fragments above 5 Mb, while SNP-array can identify submicroscopic chromosomal abnormalities that are too small to be detected by conventional karyotyping. Previous researchers found that DWS foetuses were more likely to have pathogenic copy number variations than other foetuses with neurological malformations [7, 9]. Several recent studies have also confirmed that SNP-array has outstanding contributions to the diagnosis of DWS with normal chromosomes [10–12]. It is suggested that both SNP-array and conventional karyotyping should be provided to pregnant women undergoing invasive prenatal testing due to DWS with/without other abnormal ultrasonography findings. However, not all pregnancies performed SNP-array due to the high cost in this study; a new method with low cost is needed.

Some pregnant women chose to terminate the pregnancy with DWS in ultrasonic examination due to poor prognosis, even if the karyotype analysis was normal. It has been reported that most patients with DWS have psychomotor developmental delay and mental retardation [13]. It is difficult to evaluate postnatal development according to prenatal ultrasonic examination. In this study, 4 out of 12 cases without chromosomal aberration in the nonisolated group exhibited abnormal postnatal development. However, there were no abnormalities observed after birth in 7 cases in isolated group, indicating that the prognosis of the foetus without chromosomal aberration is good in isolated DWS. However, research on gene mutations in DWS was not carried out in this study and the sample size was small. Further studies and follow-up are needed to expand the sample size.

There is no large sample size analysis of the chromosomal profile of DWS in Asian populations up to now. This study analyzed the genetic and clinical characteristics of DWS in Asian populations to enrich the understanding of DWS. Moreover, the SNP-array technique performed in this study detected several segments that may be associated with DWS, which were not previously identified, and this is important for the exploration of pathogenic regions of DWS.

## Conclusion

DWS is a major risk factor for chromosomal abnormalities, and along with other ultrasound abnormalities or advanced maternal age DWS may contribute to an increased risk of chromosomal aberrations. Pregnant women with DWS in foetal ultrasonic examination should be offered a careful and comprehensive foetal ultrasound scan and further prenatal genetic testing including karyotype analysis and SNP-array due to the high risk for foetal chromosomal abnormalities. The prognosis of the foetus without chromosomal aberration is good in isolated DWS pregnancies but poor in nonisolated DWS pregnancies.

## Data Availability

All authors had full access to the data and materials. Data is available from the authors upon reasonable request.
